# Outdoor Education in Italian Kindergartens: How Teachers Perceive Child Developmental Trajectories

**DOI:** 10.3389/fpsyg.2018.01911

**Published:** 2018-10-12

**Authors:** Francesca Agostini, Marianna Minelli, Roberta Mandolesi

**Affiliations:** Department of Psychology, Università degli Studi di Bologna, Bologna, Italy

**Keywords:** outdoor education, child development, kindergartens, longitudinal study, teachers, Italy

## Abstract

Outdoor Education (OE) refers to organized experiential education that takes place in the outdoor, characterized by action-centered and thematic learning processes. Literature shows how OE may have beneficial effects on different areas of child development, including cognitive abilities, social skills, and motor development. This relationship is not necessarily linear, but moderated by different variables. Until now, few studies have examined, using rigorous methods, the role of OE in children's development and studies of preschool aged children remain lacking. The current study aimed to explore teachers' perceptions of children's developmental trajectories over 2 school years, investigating whether teachers' perceptions differed between two kindergartens, one characterized by a consolidated OE approach and the other one characterized by a more traditional method of education. The sample was composed of 20 teachers, evaluating 93 children aged 3–5 (*M* = 46.95 months, *SD* = 6.73; 42 males): 13 teachers were from a traditional kindergarten (Traditional Group- TG) and evaluated 52 children; 7 teachers were from an OE kindergarten (Outdoor Group—OE) and observed 41 children. All the teachers completed the Kuno Beller Developmental Tables (Mantovani, 1995), in order to describe specific child developmental areas in 4 consecutive moments during 2 school years (T1-T2: January-May 2014; T3-T4: October 2014-May 2015). The 20 teachers also completed the “Outdoor Activities/Trips Diary,” an instrument created for this study to collect qualitative data on the characteristics of outdoor activities. Results showed that, in all the developmental areas, OE teachers perceived higher scores over time were found for the Outdoor Group compared to the Traditional one. Specifically, GLM ANOVAs Repeated Measures revealed a significant interaction of the 2 variables Time and Groups (*p* < 0.001): contrast analyses showed that OE children, compared to the TG children, were perceived by their teachers with higher levels in all developmental areas at T1 and T2, but not at T3 and T4. The findings suggest that the OE activites, compared to indoor ones and according to teachers' perceptions, offer greater opportunities to promote the child's development at different levels, especially when children are younger. Future studies are recommended analyzing possible moderating variables and long term effects of OE.

## Introduction

### Outdoor education: the benefits for child development

In recent years, the scientific literature in the field of pedagogy, education, developmental, and educational psychology has dedicated increasing attention to the study of Outdoor Education (OE) and its implications for child development, both on physical and psychological levels.

OE has been described as “an environment focused educational approach characterized by action-centered and thematic learning processes frequently involving outdoor activities” (Dahlgren and Szczepanski, 1998, p. 3). Higgins (1995) refers to OE as education “in” (outdoor activities), “through” (personal and social education), “about” (environmental education), and “for” (sustainability) the natural environment. These definitions emphasize the strong link between OE and the outdoor environment where the activities take place. In fact, the beneficial effects of OE on child development are substantiated by more general evidence that spending time within a natural environment offers a range of health benefits for the human being. For children, some effects may be due, at least in part, to their greater neuronal plasticity (Wells and Evans, 2003).

In detail, the non-structured and constantly changing natural context represents the ideal environment for improving child health and development. Literature has indicated that promoting outdoor play can have a significant impact on children's physical activity (Harrington and Brussoni, 2015), which in turn improves blood pressure, cholesterol, and bone density (Lewicka and Farrell, 2007; Copeland et al., 2012), contributing to the reduction and prevention of child obesity (Raustorp et al., 2012). Children are more physically active when playing outdoors. Indeed, Kneeshaw-Price et al. (2013) reported that 6- to 11-year-old children were active 41% of time outdoors compared to 18% indoors. Also, physical activity in outdoor places may lead to additional positive effects compared to indoor physical activity (Thompson Coon et al., 2011; Pesce et al., 2016), such as a lower risk for developing chronic illnesses (Strong et al., 2005) and poor mental health (Mitchell, 2013).

In addition, it has been reported that children's movement and physical activity in nature may promote favorable health behaviors and attitudes about physical fitness (Bandura, 2004; Barnett et al., 2006), by producing higher levels of physical activity in adulthood (Calogiuri, 2016).

Outdoor activities also provide the possibility of experimentation and exploration (Weber, 2010; Mahdjoubi and Akplotsyi, 2012). Exploratory behaviors in nature strengthen the locomotor and immune systems and children are therefore less prone to illness, and consequently more balanced.

Exploratory activities may also contribute to the development of self-esteem and resilience (Ceciliani and Borsari, 2009) and may foster the development of imagination and sense of wonder, promoting creative knowledge (Cobb, 1977; Dahlgren and Szczepanski, 1998; Ewert et al., 2014). In line with these findings, McAnally et al. (2018) have evaluated the effects of a 15-week outdoor education program with no access to electronic media among 14-year-old boys, reporting an improvement in creative thinking and wellbeing.

In social-relational terms, outdoor activities promote social cohesion, reduce the tendency toward conflicts and stimulate the development of a sense of autonomy and self-sufficiency (Kaplan and Kaplan, 1994; Moore, 1996). In terms of cognitive development, OE stimulates intelligence and enhances mental focus, attention, reflection, and memory (Basile, 2000; Miklitz, 2001; Hartig et al., 2003; Szcezpanski, 2007).

In primary school contexts, OE has been recognized as useful in improving peer work, enhancing leadership development, improving problem-solving skills, and reducing antisocial and deviant behaviors (Fjørtoft, 2001; Pyle, 2002; Malone and Tranter, 2003).

Despite this body of research, the literature still lacks of specificity in the investigation of outdoor benefits, especially on psychological development and mental health. Definition and operationalization of psychological constructs are not easy and the mental health outcomes are often limited to self-confidence, self-esteem, or locus of control (Gustaffson et al., 2011). Few exceptions are present in literature; for example, Gustaffson et al. (2011) investigated different areas of child mental health and showed how an OE intervention, lasting 1 year, was beneficial for children aged 6–12 years, promoting, especially in boys, a decrease in different mental health problems. Furthermore, a previous study by two of the authors of this paper (Monti et al., 2017) showed positive effects of a 1-year OE intervention in nursery schools, for children aged 1–3 years: compared to children in more traditional nursery schools, following daily OE activities children showed greater improvements in cognitive, social and emotional development, motor skills, and body functions (e.g., breathing, digestion, sleeping). A study by Ulset et al. (2017) followed a cohort of 562 Norwegian children aged 3 to 7 years and measured different mental health dimensions, finding that inattention-hyperactivity symptoms tended to decrease and short-term memory (as measured by a digit span task) tended to improve as time spent outdoors in school increased.

### Outdoor education and child development: moderating variables and the role of teachers

Considering the beneficial influence of an OE approach on child development, it is relevant how OE is implemented in daily routines in educational contexts. Many European and non-European countries have included OE in daily activities in nurseries, kindergartens, and primary schools. For instance, in Scandinavian countries, which highly value children's outdoor play and activities as a relevant part of daily lives (Norðdahl and Einarsdóttir, 2015), many kindergartens offer high quantities of outdoor activities (Borge et al., 2003; Nilsen, 2008).

However, it is not simple to implement OE and the relationship between OE and child development outcomes is not necessarily linear. Indeed, different factors may influence this relationship and some of them may act as moderators, including child's gender, child temperament, family socioeconomic status, and parents' mental health (Ulset et al., 2017). Also, variables concerning the kindergarten or day-care center may play the role of moderating factors, such as group size and teacher-child ratio, barriers related to the natural context and/or the architectural structures (Ulset et al., 2017), and availability of specific play objects and materials in outdoor places (Brown et al., 2009).

Other important variables that may moderate the relation between activities in outdoor places and promotion of children's development include the quality of the child-teacher relationship (Tonge et al., 2017) and the parents' and teachers' perceptions and beliefs about the importance of the outdoor environment and OE (Insenberg, 1990; Kagan, 1992; Pjares, 1992; Fang, 1996). As far as parents are concerned, it has been shown that they usually understand the benefits of natural play spaces, appreciating natural environments much more than urban ones for their children's activities and learning (Wang et al., 2018). Regarding teachers' perspectives McClintic and Petty (2015), have explored the beliefs about preschool outdoor play, reporting that teachers interviewed in their study considered outdoor play essential along with the children's opportunity to experience free play. However, they perceived their role as limited to supervising children's activities and they did not fully understand the potential of the outdoor environment for child development.

Teachers have the tasks of planning activities, providing challenging and creative environments, supporting child strengths, all while remaining attuned to children's needs and avoiding disrupting or interrupting their activities (Wilford, 1996; Frost et al., 2011). Therefore, the ways that teachers explain and propose OE activities to children, recognize their natural need to move and experiment and support their attempts, sensorial experiences, and actions in the outdoor context are critical to success (Nelson, 2006; Gehris et al., 2014). However, OE is rarely held as a priority by many teachers (McClintic and Petty, 2015) and they tend to give less time and attention to outdoor activities compared with indoor activities (Wellhousen, 2002).

Based on this, the research exploring teachers' perceptions following implementation of OE is essential but still poor. There is a need to investigate more accurately whether and how teachers perceive the usefulness of OE to foster child development. It is also relevant to investigate how they promote outdoor activities, structure play and outdoor environments for different child age ranges, according to different environmental places (Hu et al., 2015).

### The current study

The current study aimed to explore teachers' perceptions of children's developmental trajectories over 2 school years, comparing a kindergarten with an OE approach and a kindergarten with a traditional education approach (that is, using the outdoor environment only as a recreational space).

The reasons for choosing preschool age were the following: (a) during this time period, children's development is characterized by acquisition of skills such as symbolic play, differentiation of imaginary vs. real, theory of mind, story-telling, counting, and eating independently (Sheridan, 2008); (b) Outdoor Education in Italy is more frequently applied in kindergartens compared to primary schools.

More specifically, the research questions posed in this study were developed based on the literature evidence that OE contributes to motor, cognitive, social, and emotional skills development beginning in early childhood. Furthermore, research questions were developed based on a previous study by two of the authors (Monti et al., 2017), comparing OE vs. traditional educational approach in 1 to 3 year-olds in nursery schools.

Based on the results from this study, we hypothesized that, according to teachers' perception, children aged 3–5 years old attending an OE kindergarten would demonstrate greater improvement in development compared to children in a traditional kindergarten. Second, we aimed to investigate if the teachers' perception about child development would change or remain stable across a wide period of time, so we collected different time assessments during 2 consecutive school years.

We also aimed to explore the characteristics of outdoor activities in both kindergartens, e.g., duration, daily weather, and type of activity: we expected teachers from the OE kindergarten to show a greater tendency to go and stay outdoors during the 2 years, both in terms of frequency and duration of outdoor activities, and also with a different psycho-educational quality of the time spent outdoors.

## Materials and methods

### Participants

The total sample included 20 teachers working at the two kindergartens: 13 teachers worked in the kindergarten adopting traditional educational activities and represented the Traditional Group (TG), while 7 teachers worked in a kindergarten applying a continuous OE program, representing the Outdoor Education Group (OE). These 7 teachers took part in the same training in OE, characterized by a 15-day intensive training in an international Outdoor Education and Learning Centre (Sweden) and 1-year continuous training in Italy. Characteristics of the teachers in terms of years of experience in teaching are shown in Table 1; no significant differences were found between the two groups of teachers (*p* > 0.05).

**Table 1:** Descriptive characteristics for teachers and children.

**Teachers**	**OE group (*N* = 7)**	**Traditional group (*N* = 13)**
Years of teaching, mean (SD)	8.57 (4.35)	13.61 (9.83)
Years of experience in OE, mean (SD)	8.71 (3.4)	–
**Children**	**OE group (*****N*** = **41)**	**Traditional group (*****N*** = **52)**
Mean age (SD)	47.20 (6.52)	46.75 (6.95)
Gender, males (%)	13 (31.7)	29 (55.7)

During the two school years, the teachers evaluated 230 children aged 3–5 years (*M* = 48.7 months, *SD* = 10; 119 males). Based on the aims of this study, only the children with 4 complete evaluations across the 2 years were considered. Therefore, the total sample of children was 93, aged 3–5 years (*M* = 46.95 months, *SD* = 6.73; 42 males). Specifically, the TG teachers evaluated 52 children (*M* = 46.75, *SD* = 6.95; 29 males), and the OE teachers observed 41 children (*M* = 47.20, *SD* = 6.52; 13 males) (Table 1). The children attending the two kindergartens were homogenous in terms of age, gender, and socioeconomic status. Cases of social or developmental risk were not included in the study sample.

The present study was approved by the Directors and the Teachers' Colleges of the two kindergartens, in accordance with the recommendations of school rules. Children's parents were informed about the research and volunteered their child's participation in the study, providing the written informed consent in accordance with the Declaration of Helsinki.

### Procedure

The study was conducted between January 2014 and May 2015 and involved two kindergartens of Emilia Romagna region, in the North of Italy. One kindergarten was chosen because the teachers were experienced and trained in the Outdoor Education approach (OE Group) and OE represented a daily routine. The other kindergarten was characterized by a more traditional educational approach (Traditional Group) and the teachers were not trained in OE.

All the teachers involved in the study completed the Kuno Beller Developmental Tables (Mantovani, 1995) in four consecutive moments during 2 school years (T1-T2: January-May 2014; T3-T4: October 2014-May 2015). The teachers were specifically trained in the use of the Kuno Beller Developmental Tables before starting the data collection; two psychologists with expertise in using this tool held this training, which was characterized by explanations of the items, coding of children's behaviors, and accurate supervision. The training period lasted 1 month in both kindergartens for all the teachers involved in the research.

In addition to the Kuno Beller Developmental Tables, the teachers completed the “Outdoor Activities/Trips Diary” every time they went outdoors with their classes.

### Measures

#### Child development

All the 20 teachers completed the Kuno Beller Developmental Tables (Mantovani, 1995), in order to describe children's development according to specific areas. The Kuno Beller Developmental Tables represent a useful instrument to collect adults' (i.e., parent or teacher) perception of child development and to plan educational activities. The Kuno Beller includes the following 8 developmental areas: *Domain of Body Function, Awareness of the Surrounding Environment, Social and Emotional Development, Play, Language, Cognitive Development, Gross* and *Fine Motor Skills*. In particular, *Domain of Body Function* collects the progressive perception of the self, the child's autonomy, in terms of physical care and many body functions (e.g., sleeping, eating), while *Awareness of the Surrounding Environment* defines the progressive awareness the child has of the surrounding world.

In order to complete the Kuno Beller Developmental Tables, the adult starts answering a detailed list of items from the development phase in which the child does all the things described and stops in the phase where s/he does not see any behavior depicted. This is repeated for all the eight developmental areas. In order to complete the Kuno Beller, parents or teachers have to report what children do in daily situations, therefore the more they observe the child the more the answers to the test will be accurate. As a result, the instrument allows obtaining a picture of the level of child development for every developmental domain and of the relationships among the different domains.

#### Outdoor activities

The “Outdoor Activities/Trips Diary” is an instrument created for the purposes of this study to collect qualitative data on the characteristics of outdoor activities. Specifically, the teachers used it for the 2 years after each trip, answering the following items: *Period of the year, Daily Weather* (sunny, cloudy, foggy, light rain, snow, windy), *Place* (schoolyard, park, urban space, other), *Group* (small, max. 5 children; big, max. 10 children; whole class), *Duration* (minutes < 30, 30–60, 60–120, >120), and *Activity* (free play, guided play, free exploration, guided exploration, physical education, guided trip, other).

With regard to *Place*, the schoolyard of the two kindergartens had different characteristics: the yard of the OE kindergarten consisted of a green park with some centuries-old trees (e.g., firs, willows, maples), plants and flowers, and without any play structures. The traditional schoolyard contained grass and cement without larger plants, trees, and play structures.

With regard to *Activity*, “free play” means children play games they choose and with materials they want to play with. In contrast, “free exploration” means teachers give one simple instruction to children regarding a specific explorative task to do and children decide how to perform it. For instance, the teacher may give children magnifying lenses, suggesting that they look for ants, but letting them decide how to do it. “Guided exploration” includes teachers giving several instructions to children in order to perform a specific explorative task. “Guided trip” consists of a school trip with a specific aim that is shared with children at the moment of planning the trip (e.g., visiting a public park outside the kindergarten, or visiting an aquarium in town). The category of activity called “other” consists of all the activities not included in previous categories.

### Statistical analyses

Mixed-Model Repeated Measures analysis of variance (ANOVA) was used to analyze changes in children's development across the 4-time-points assessments in all the selected Kuno Beller domains for both groups, considering as independent variables: group (Outdoor vs. Traditional), period (T1, T2, T3, T4), and gender (males vs. females). In order to explore the characteristics of the outdoor activities for both the OE and Traditional groups, descriptive analyses were performed on the data from the Outdoor Activities/Trips Diary. Data analyses were run using the Statistical Package for Social Sciences (S.P.S.S.), version 21.0.

## Results

### Kuno beller developmental tables

Descriptive statistics of Kuno Beller Developmental Tables are reported in Table 2.

**Table 2:** Descriptive statistics of Kuno Beller developmental tables.

**Domains**	**OE group (*****N*** = **41) Mean scores (SD)**				**Traditional group (*****N*** = **52) Mean scores (SD)**			
	**T1**	**T2**	**T3**	**T4**	**T1**	**T2**	**T3**	**T4**
Body function	11.02 (0.81)	11.53 (0.77)	12.16 (0.71)	12.81 (0.71)	10.15 (1.03)	11.01 (1.22)	11.96 (0.94)	12.39 (1.24)
Awareness of surrounding environment	11.35 (1.22)	11.75 (0.93)	12.30 (0.91)	13.20 (0.66)	10.07 (1.80)	11.17 (1.37)	12.03 (1.11)	12.86 (1.09)
Social and emotional development	11.18 (1.09)	11.72 (0.67)	12.22 (1.01)	12.96 (0.94)	10.24 (1.14)	11.03 (1.31)	12.22 (1.03)	12.86 (0.94)
Play	11.26 (1.08)	11.87 (0.57)	12.35 (1.14)	13.15 (0.99)	9.89 (1.22)	10.88 (1.43)	11.88 (1.24)	12.78 (1.14)
Language	11.01 (1.30)	11.50 (0.73)	12.01 (1.13)	12.88 (1.03)	9.83 (1.53)	10.87 (1.48)	11.80 (1.37)	12.74 (1.24)
Cognitive development	10.94 (0.89)	11.30 (0.54)	11.78 (0.82)	12.49 (0.95)	9.63 (1.35)	10.59 (1.40)	11.43 (1.28)	12.58 (1.31)
Gross motor skills	11.79 (1.01)	11.99 (0.76)	12.72 (1.04)	13.32 (0.80)	10.87 (0.91)	11.74 (1.24)	12.37 (1.05)	12.96 (1.07)
Fine motor skills	10.86 (0.76)	11.34 (0.77)	11.86 (0.56)	12.73 (0.88)	10.01 (1.34)	10.72 (1.46)	11.73 (1.39)	12.56 (1.28)

*T1, January 2014; T2, May 2014; T3, October 2014; T4, May 2015*.

Results from Mixed-Model Repeated Measures analysis of variance (ANOVA) showed no significant gender differences. However, a significant main effect of Time (*p* < 0.001) on children's development was found, suggesting that children from both groups progressively increased their skills from T1 to T4, according to their teachers' perspective. This was observed for all eight developmental areas as measured by Kuno Beller: *Domain of Body Function, Awareness of the Surrounding Environment, Social and Emotional Development, Play, Language, Cognitive Development, Gross* and *Fine Motor Skills*.

**Figure 1:**
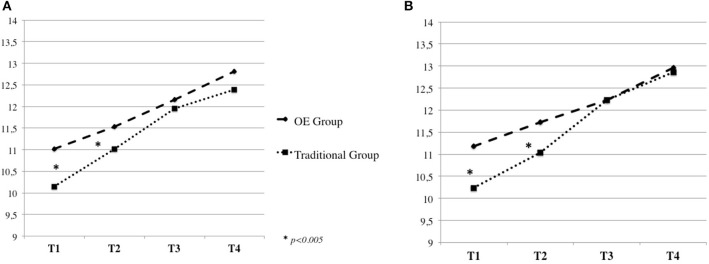
Kuno Beller domain of body function **(A)** and social and emotional development **(B)**.

**Figure 2:**
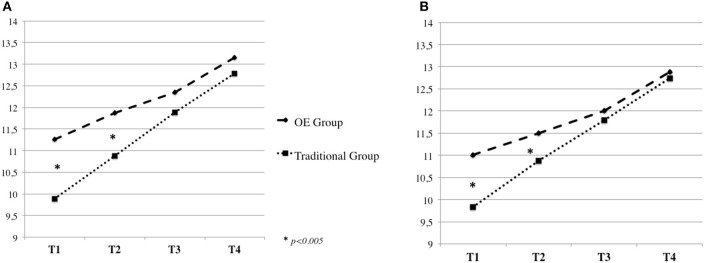
Kuno Beller play **(A)** and language **(B)**.

**Figure 3:**
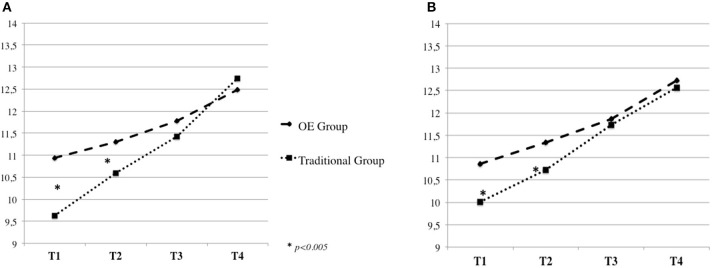
Kuno Beller cognitive development **(A)** and fine motor skills **(B)**.

In addition, significant Time by Group interactions were found for the eight Kuno Beller domains (all *p* < 0.005), indicating that children's development differed over time between the OE and the Traditional groups (Table 3). Specifically, contrast analyses revealed significant linear interactions, with children in the OE Group showing, at T1 and T2, significantly higher mean values compared to the Traditional Group in the following developmental areas: *Domain of Body Function* [*F*_(1, 91)_ = 6.99, *p* = 0.010] (Figure 1A), *Social and Emotional Development* [*F*_(1, 91)_ = 14.83, *p* = 0.000] (Figure 1B)*, Play* [*F*_(1, 91)_ = 18.27, *p* = 0.000] (Figure 2A)*, Language* [*F*_(1, 91)_ = 19.15, *p* = 0.000] (Figure 2B), *Cognitive Development* [*F*_(1, 91)_ = 32.23, *p* = 0.000] (Figure 3A)*, Fine Motor Skills* [*F*_(1, 91)_ = 16.49, *p* = 0.000] (Figure 3B).

**Table 3:** Results from mixed-model repeated measures ANOVA: Values for linear Time X Group interactions.

**Period X Group**	**df**	***F***	***p***	**η*_*p*_*^2^**
Kuno domain of body function	1	6.99	0.010	0.27
Kuno awareness of surrounding environment	1	8.98	0.004	0.30
Kuno social and emotional development	1	14.83	0.000	0.38
Kuno play	1	18.27	0.000	0.41
Kuno language	1	19.16	0.000	0.42
Kuno cognitive development	1	32.23	0.000	0.51
Kuno gross motor skills	1	5.49	0.021	0.24
Kuno fine motor skills	1	16.49	0.000	0.15

**Figure 4:**
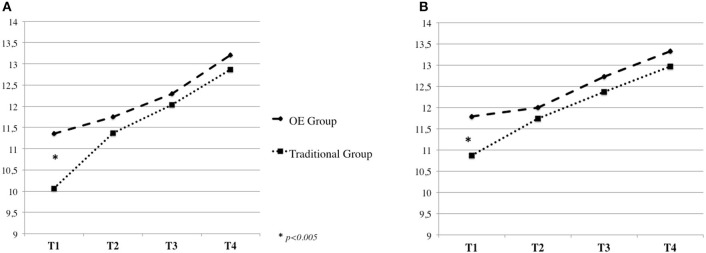
Kuno Beller awareness of surrounding environment **(A)** and Gross Motor Skills **(B)**.

In *Awareness of Surrounding Environment* [*F*_(1, 90)_ = 8.98, *p* = 0.004] (Figure 4A) and *Gross Motor Skills* [*F*_(1, 90)_ = 5.49, *p* = 0.021] (Figure 4B), children in the OE Group showed significantly higher mean values compared to children of the Traditional group, but only at T1.

### Outdoor activities/trips diary

**Figure 5:**
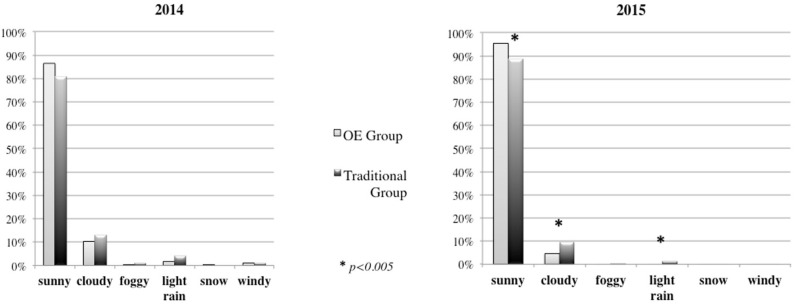
Weather conditions during outdoor activities in 2014 and 2015.

Results showed that children in the OE kindergarten went outdoors more frequently compared to the children in the traditional kindergarten: 467 times compared to 176 in 2014; 522 times vs. 236 in 2015. In analyzing the characteristics of the outdoor activities completed by both groups, we did not find any differences regarding the *weather conditions* in 2014 (Figure 5), as both groups went outdoors more frequently during a “sunny day” rather than during other weather conditions. During 2015, the OE Group went outdoors more frequently on sunny days compared to the Traditional Group; in contrast, on cloudy or lightly raining days, the Traditional Group went outside with higher frequency than the OE group.

**Figure 6:**
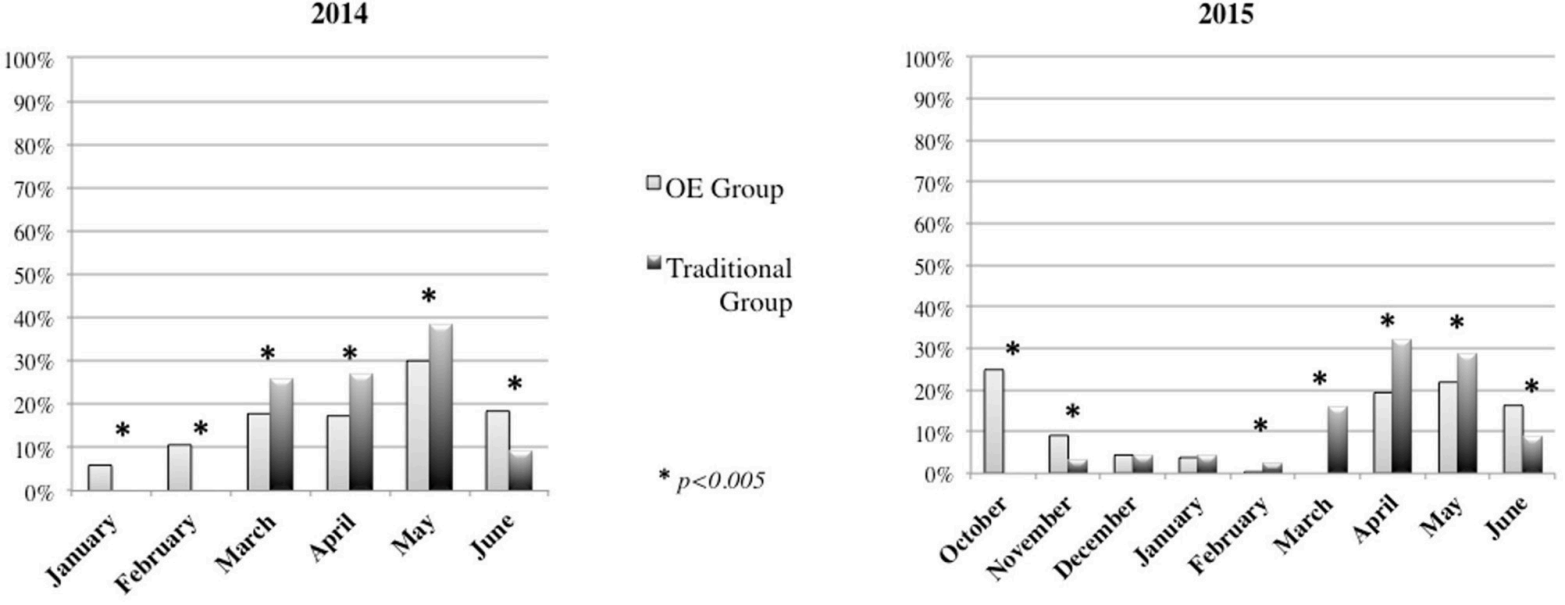
Outdoor activities during different months in 2014 and 2015.

The OE Group, in 2014, went out more frequently compared the Traditional Group during January, February, and in June, while the Traditional Group went out more frequently during March, April and May (Figure 6) (χ^2^ = 48.318, *p* = 0.0005). Similarly, during 2015, the OE Group went outdoors more frequently during October, November, and June, while the Traditional Group preferred to go outdoors in February, March, April, and May (Figure 6) (χ^2^ = 181.532, *p* = 0.0005).

**Figure 7:**
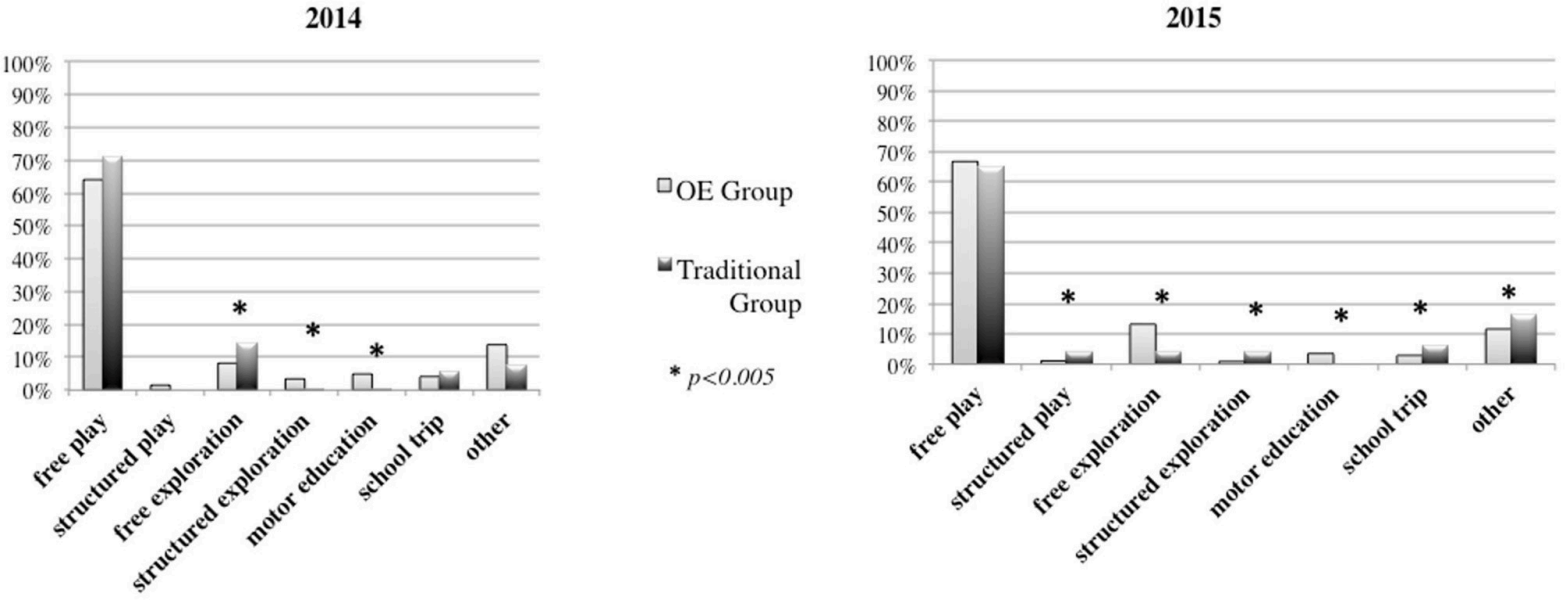
Types of outdoor activities in 2014 and 2015.

Regarding the *types* of activities, in 2014 the OE Group more frequently chose physical education and structured exploration, while the Traditional Group preferred to go outside for free exploration (Figure 7) (χ^2^ = 23.820, *p* = 0.001). In 2015, the OE Group more frequently chose physical education and free exploration, while the Traditional Group preferred the school trip, structured play, structured exploration and “other” types of activities (Figure 7) (χ^2^ = 55.020, *p* = 0.0005).

Analysis of types of activities in relation to seasons of the year demonstrated that in the spring of 2014 (March and April) the OE Group, as compared to the Traditional Group, had a stronger preference for structured exploration and physical education (χ^2^ = 13.657, *p* = 0.018). During summer 2014 (May and June), we did not find any significant differences between the two groups (*p* > 0.05). During fall 2014 (October and November), the OE Group showed a greater preference for free play compared to the other group, while the other group displayed a greater preference for structured exploration (χ^2^ = 48.818, *p* = 0.0005). During winter (December 2014-February 2015), the OE Group showed a greater preference for free play and free exploration, while the Traditional Group more frequently used structured play (χ^2^ = 10.426, *p* = 0.034). During spring 2015 (March and April), the OE Group more frequently engaged in free exploration and physical education, while the Traditional Group preferred free play, structured play, and structured exploration (χ^2^ = 36.863, *p* = 0.0005). During summer 2015 (May-June), the OE Group showed a greater preference for free exploration and physical education, while the Traditional Group preferred free play (χ^2^ = 16.165, *p* = 0.006).

**Figure 8:**
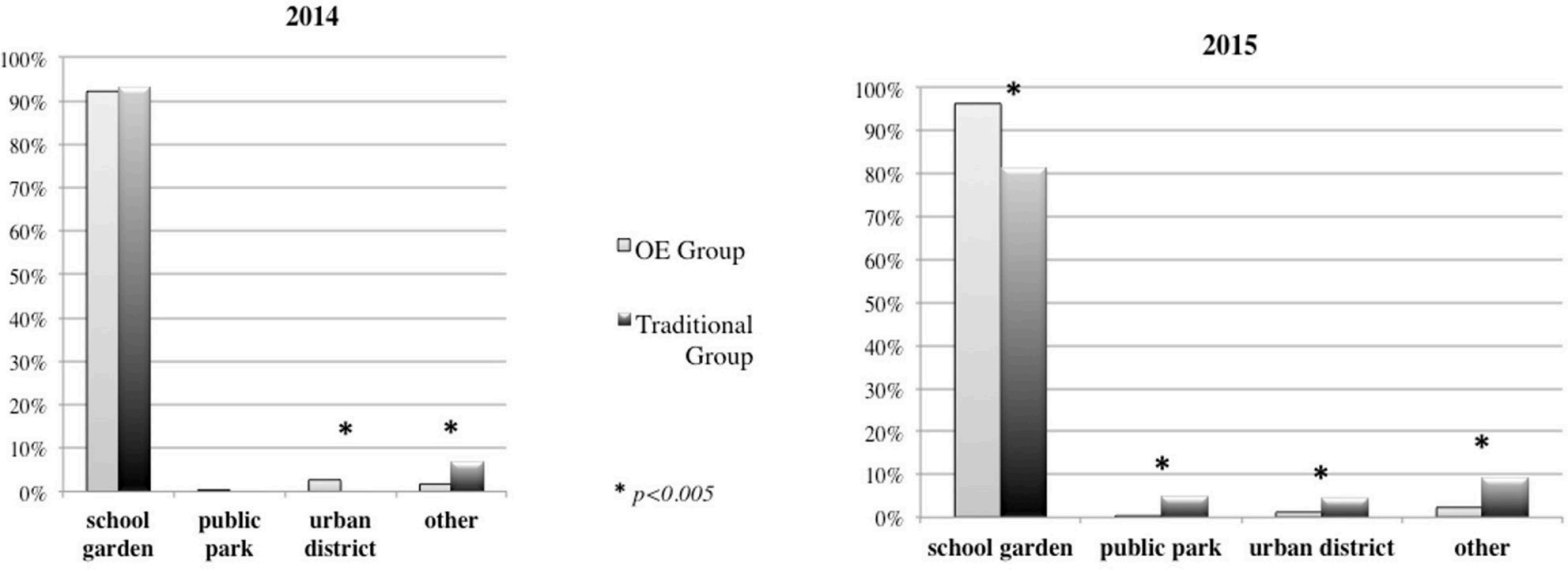
Space used for outdoor activities in 2014 and 2015.

Some differences emerged regarding the *places* used for outdoor activities. In 2014, the OE Group more frequently used the urban district compared to the Traditional Group, (Figure 8) (χ^2^ = 21.745, *p* = 0.000). In 2015, the OE Group mainly chose the schoolyard, while the Traditional Group more frequently used the public park, the urban district and “other” places (Figure 8) (χ^2^ = 49.409, *p* = 0.0005).

**Figure 9:**
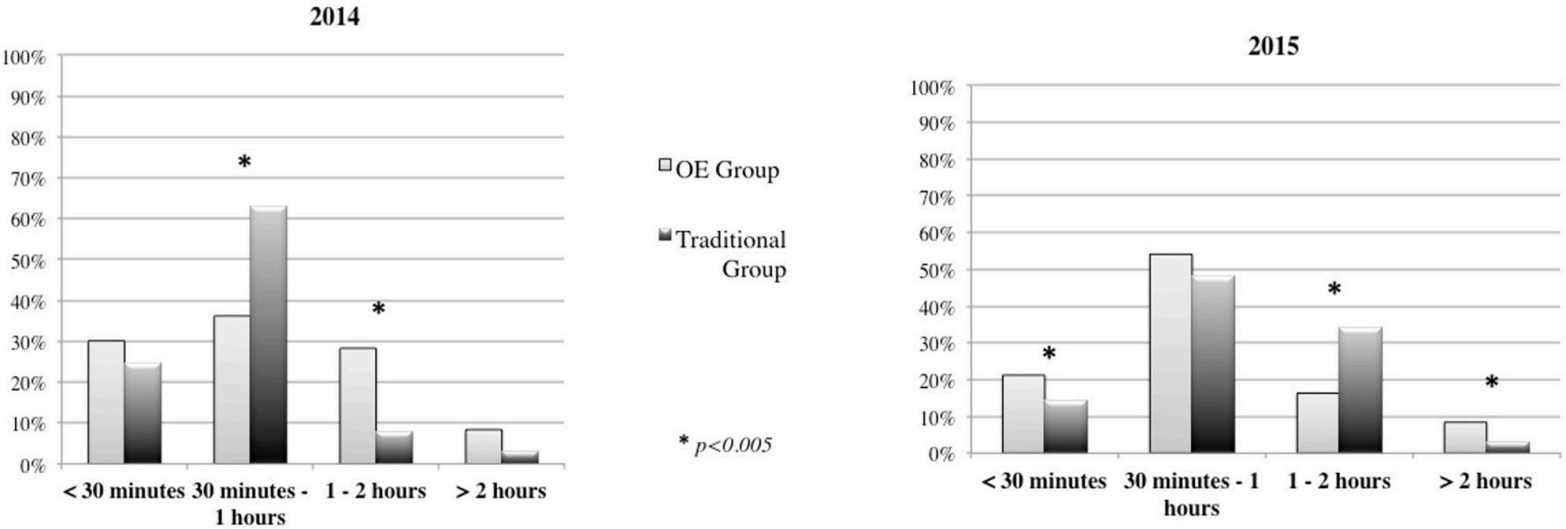
Duration of time for each outdoor activity in 2014 and 2015.

Finally, by looking at the variability of *time duration* for outdoor activities, we see that in 2014, the OE Group preferred to spend 1–2 h outdoors compared to the Traditional Group, which preferred to spend 30 min−1 h outdoors (Figure 9) (χ^2^ = 45.298, *p* = 0.000). In contrast, in 2015 the OE Group showed greater variability, preferring to spend less than 30 min or more than 2 h outdoors, while the Traditional Group tended to spend 1–2 h outdoors (Figure 9) (χ^2^ = 34.010, *p* = 0.000).

## Discussion

Literature has emphasized the potential benefits of OE for children's well-being and development, due to a joint effect of enhanced physical activity and being in a natural environment (Gustaffson et al., 2011). To promote these effects, teachers play a key role when implementing OE activities in kindergartens and adapting them to children's ages. Notwithstanding this, the literature has not sufficiently explored, up to now, how teachers perceive OE and its benefits on child development.

The main aim of this longitudinal study was to investigate how teachers perceived children's development over 2 consecutive years, comparing a kindergarten characterized by a definite OE approach to a kindergarten with a traditional educational method. Specifically, we aimed to analyze whether teachers' perceptions about child development were different according to the kind of educational method (OE vs. traditional). Second, we aimed to explore whether the teachers' perception remained stable or changed across the 2-year period of observation. Third, we aimed to investigate how teachers implemented outdoor activities in the two kindergartens.

Main results derived from the Kuno Beller Tables showed that for all eight child developmental domains, there was a significant interaction between time of assessment and group condition. This means that perceptions about child development were significantly more positive for OE teachers compared to the teachers using a traditional approach, but this was only true for the assessments at T1 and T2, not at T3 and T4, due to the converging flatter slope of the OE condition and steeper slope of the traditional condition. In other words, older children, independent from the educational method, showed similar levels of development according to their teachers' perception.

In interpreting these results, several factors must be taken into consideration. First of all, when the first assessment was conducted (January 2014), the two groups of children were already showing, according to the teachers' perception of developmental characteristics, a significant difference in developmental level. We did not have the opportunity, due to constraints in the implementation of the study, to assess children's development before the children started kindergarten in order to establish whether there were pre-existing differences in developmental level. However, we are confident that the two groups of children did not differ according to measured, common socio-demographic characteristics. We also know that the OE teachers were using the daily OE activities since the beginning of the school year (September 2014).

Therefore, while we do not have a measure at baseline, we may hypothesize the following scenarios to explain the converging developmental trajectories observed in the two groups, one assuming that the children in the two groups were developmentally similar at the beginning of the school year, the other for a scenario in which the two groups had unmeasured, pre-existing differences in developmental level.

If the two groups were developmentally similar at the beginning of the school year, OE may have been quite beneficial for children's development early in the school year before data collection began. This initial developmental rate impact, if present, does not appear to sustain its pace over the next year as the rate of development (slope of the line) is flatter than for the Traditional Group, eventually intersecting with the developmental trajectory of the Traditional Group. It may be that OE is more effective for younger children compared to older ones; there may be more sensitive periods for the benefits of OE on child development, as already shown by a previous study (Monti et al., 2017). We also have to consider that teachers may be more prone to perceiving children's improvement when they are younger, as children are developing very rapidly and developmental progress may be more obvious on the Kuno Beller Table scores at younger ages than at older ages. A second interpretation is that OE has its strongest impact earlier in an intervention, regardless of at what age that intervention takes place. The slowing of the rate of development demonstrated by the OE group may therefore reflect diminishing strength of impact of OE in later stages of the intervention.

If the groups of children were already developmentally different at the beginning of the school year (as a result of unmeasured influences of demographic characteristics, for example), what we observed across the 2 years seems to reflect a slower developmental trajectory for the children in OE, possibly reflecting a ceiling effect. It is also possible that traditional kindergarten education is more effective for students of lower developmental level, reflected in a steeper trajectory, than OE is for students of higher developmental level, reflected in a flatter trajectory.

It is also important to remember that we were measuring teacher perceptions of children's development. As we reported in the aims of the study, we specifically wanted to focus on teachers' perceptions based on the perspective, evidenced by the literature, that their perceptions and attitudes, educational experiences and teaching have a significant impact on children's wellbeing, learning and development. We are aware that teachers' perceptions may be influenced by a host of variables and are not fully comparable to child development as observed by direct measures. Notwithstanding, OE teachers received the same training in the use of the Kuno Beller as did traditional kindergarten teachers. In addition, this instrument is characterized by items measuring the presence/absence of specific behaviors (e.g., the child is able to count up to 20) and is therefore based on objective benchmarks.

In our study, teacher perceptions of children's development are in line with the main evidence from the literature concerning the beneficial effects of activities in nature for children. Wardle (1997) has demonstrated that physical activities in nature help foster children's communication, emotional, social, and cognitive skills, not just motor skills. Gill (2014) has underlined how, in the outdoor environment, the child is facilitated in establishing a connection between his/her individual sensory experiences, motor activities and learning; also, his/her cognitive processes can be enhanced, with positive consequences for motor development, social skills, language, and communication, among others. In the more optimistic interpretation of the data explained above (no pre-existing developmental differences; strong initial impact of the OE intervention), these results may suggest that continuous outdoor activities provide greater opportunities for teachers' attitudes to promote children's development; this may occur when teachers perceive the natural environment as an educational and developmental setting rather than only a recreational one. When teachers hold this view about the outdoor context, specific learning experiences may be achievable.

Our results would indeed suggest how OE activities may promote an improvement in development at many different levels, at least in the short term. Similar results were obtained by a previous study with a similar research design (Monti et al., 2017): in this case, children attending nursery schools using OE showed a significant improvement in all the developmental domains (as measured by Kuno Beller) after 1 year of OE intervention, compared to a group of children following a traditional educational approach.

When analyzing the data collected through the Outdoor activities/diaries in the present study, some interesting results emerged regarding the psycho-educational quality of the activities undertaken in each kindergarten. First, the most evident result was that children from the OE kindergarten were going out for significantly more time than the children in the other kindergarten during the two school years, specifically more than twice as much time. It was clear that the children in the OE kindergarten took more advantage of the outdoors during the autumn and winter compared to children attending the traditional kindergarten. Also, a difference emerged regarding the spaces used for activities: while the OE kindergarten had a schoolyard and this represented the most used space during the 2 years, the teachers in the traditional kindergarten had to take advantage of more urban spaces or other places outside the school campus because they did not have an appropriate schoolyard. We may hypothesize that this partly influenced the kind of outdoor activities chosen, because the OE children were spending more time, somewhat dependent on the year and the season, in activities that could be easily experienced in the schoolyard, such as physical education and free/structured exploration; on the contrary, the children attending the traditional kindergarten were more frequently experiencing structured activities outside the school, always depending on the year and season. The difference in the outdoor activities was evident also regarding the duration of outdoor activities, as the OE teachers tended to go outside for longer periods, compared to those in the traditional kindergarten.

These results support and confirm the differences in usage of outdoor space by the OE teachers vs. the teachers in the traditional setting. Children show a spontaneous preference for being outside than inside and a desire to use the outdoor environment at school exploring things and enjoying what they can find in the outdoors (Norðdahl and Einarsdóttir, 2015). The activities proposed by OE teachers seem to be more in line with children's preferences. OE teachers have the possibility of proposing to children activities such as physical education and play, both free and structured. We have to remark that teachers play a key role in prompting children in play. Play is a fundamental activity and method of self-expression for children in the 3–5 year old age range and supports the child's development and his/her experience in making sense of the world (Soini et al., 2014). Literature shows that more playful activities (e.g., exploration) are associated with benefits related to physical activity and mental and emotional health (Gill, 2014). Also, free play has frequently shown significant positive effects on cognitive and social-emotional development, independence and creativity (Frost et al., 2011). Our results would seem to support these positive effects: in the first year, OE children showed, above all, higher levels of development than the traditional kindergarten children (including motor development, social, and emotional development, play).

Also, prior research suggests that less playful activities (such as field trips) are more associated with educational benefits than health benefits (Gill, 2014). Therefore, it would seem that the kind of outdoor activities proposed by teachers in the traditional kindergarten also promoted child development but not in the same manner as the activities prompted by OE teachers.

Some limitations of the study need to be acknowledged. The most important ones have been already reported: it was not possible to measure the children's development at baseline and we did not add any measure of child development rated by external observers, due to restrictions set by the school directors. Second, the teachers working in the OE kindergarten already had a high level of expertise in OE and had become accustomed to working with this kind of approach over a number of years. A more rigorous research design would have required introducing the OE intervention starting from similar baseline conditions in both kindergartens, to increase the validity of the teachers' evaluations. Third, for reasons of the same school restrictions, we could not gain access to other demographic information about the children, their families and the teachers. Therefore, since it was not possible to run statistical analyses for exploring the role of these data as possible moderating variables, the validity of our results needs to be confirmed by future studies. Fourth, in the period Feb-Mar 2015, due to maintenance work, the yard of the OE kindergarten could not be properly used, so this could have influenced the data collected. Finally, the sample included only two kindergartens; a replication of the study with a larger sample size is recommended. In summary, further research is needed comparing Kuno Beller Tables with more objective measures of child development, specifically exploring the possible benefits of OE on different age ranges, as well as the sustainability of impact of OE over time.

Notwithstanding these limitations, this study has several strengths: within this research field, where literature seems to prefer cross-sectional designs (Gustaffson et al., 2011), this study presents a longitudinal design, with a long time period of observation (2 years), including four follow-ups. Also, even if we used only one instrument, we chose a robust, objective tool, exploring in detail specific areas of child development connected to mental and physical health dimensions. Lastly, all the teachers received appropriate and rigourous training before utilizing the tool and they reported, after the use of Kuno Beller Tables, to have gained more awareness of the relevance of child development observations for their daily work.

## Conclusion

A high frequency of outdoor activities in kindergartens represents a practical, easy, effective and cheap way to support child development (Ulset et al., 2017). OE offers a “complex learning environment where nature-based learning is being embraced by educators and can be seen in the experiences offered to children” (p. 11, Macquairre et al., 2015). How teachers perceive the natural environment and the benefits of OE are key factors for the implementation of daily outdoor activities with positive effects on child development. Teachers planning appropriate and creative use of the outdoors, in fact, support the promotion of children's well-being and mental health. Children in OE kindergartens seem to significantly take advantage of this educational approach, as they have a greater opportunity, compared to children attending more traditional kindergartens, to experience continuous and multiple OE activities during the school years, with more benefits at least in the short term.

For these reasons, social policies should engage more resources to spread OE practices starting from early childhood. At the same time, further research should be conducted to investigate the benefits of OE at different child age ranges, including the role of moderating variables, as well as the sustainability of impact beyond the short term.

## Author contributions

FA was the leading author in conceptualizing and coordinating the research design and writing the manuscript. MM contributed to all the steps of the study and to writing and revising the manuscript. RM contributed to the data coding, writing and revision of the manuscript. All the authors accepted accountability for the final version of the manuscript.

### Conflict of interest statement

The authors declare that the research was conducted in the absence of any commercial or financial relationships that could be construed as a potential conflict of interest. The reviewer SR and handling Editor declared their shared affiliation.
